# Optimization of Isocyanate Content in PF/pMDI Adhesive for the Production of High-Performing Particleboards

**DOI:** 10.3390/polym15244645

**Published:** 2023-12-08

**Authors:** Jakub Kawalerczyk, Dorota Dukarska, Mateusz Barczewski, Dorota Dziurka, Radosław Mirski

**Affiliations:** 1 Department of Mechanical Wood Technology, Faculty of Forestry and Wood Technology, Poznan University of Life Sciences, Wojska Polskiego 38/42, 60-627 Poznan, Poland; dorota.dukarska@up.poznan.pl (D.D.); dorota.dziurka@up.poznan.pl (D.D.); radoslaw.mirski@up.poznan.pl (R.M.); 2 Institute of Materials Technology, Poznan University of Technology, Piotrowo 3, 61-138 Poznan, Poland; mateusz.barczewski@put.poznan.pl

**Keywords:** PF/pMDI adhesive, particleboard, isocyanate content, hybrid resin

## Abstract

Due to the fact that impregnation with fire retardant usually reduces the strength of the produced particleboards, this research was carried out to investigate whether it is possible to use phenol–formaldehyde (PF) resin modified using various amounts (0%, 5%, 10%, 15%, and 20%) of polymeric 4,4′-methylene diphenyl diisocyanate (pMDI) for this purpose. The need to optimize the addition of pMDI is particularly important due to health and environmental aspects and high price. Furthermore, the curing process of hybrid resins is still not fully explained, especially in the case of small loadings. Manufactured particleboards differed in the share of impregnated particles (50% and 100%). The mixture of potassium carbonate and urea was used as the impregnating solution. Based on the outcomes of hybrid resins properties, it was found that the addition of pMDI leads to the increase in solid content, pH, and viscosity of the mixtures, to the improvement in resin reactivity determined using differential scanning calorimetry and to the decrease in thermal stability in the cured state evaluated using thermogravimetric analysis. Moreover, particleboard property results have shown that using impregnated particles (both 50% and 100%) decreased the strength of manufactured boards bonded using neat PF resin. However, the introduction of pMDI allowed us to compensate for the negative impact of fire-retardant-treated wood and it was found that the optimal loading of pMDI for the board containing 50% of impregnated particles is 5% and for board made entirely of treated wood it is 10%.

## 1. Introduction

Over the years, adhesives have played and still continue to play a huge role in the effective use of wood resources in various forms [1]. For this purpose, the binding properties of some natural polymers have been known and used for centuries in the production of wooden objects. However, the rapid development of plastics chemistry in the 20th century resulted in the development of more cost-effective synthetic adhesives that behaved better in humid conditions [2,3]. The most commonly used thermosetting, formaldehyde-based, polycondensation adhesives include urea–formaldehyde (UF) resin, melamine–urea–formaldehyde (MUF) resin and phenol–formaldehyde (PF) resin, and their development is the key factor for the production of functional wood-based products [4]. These adhesives gradually took over the market and are currently estimated to account for approx. 95% of all adhesives applied in the wood-based materials industry [5,6]. Therefore, considering their dominant market share and the growing requirements in terms of the performance of wood-based materials, studies on the possibility of their enhancement are still the subject of numerous scientific works conducted worldwide [7,8,9].

Research on the formaldehyde-based adhesives modified using pMDI (polymeric 4,4′ -methylene diphenyl diisocyanate) has shown some promising results in recent years. Isocyanates as wood adhesives stand out because of their high adhesion and cohesion strength. Moreover, created bonds are characterized by significant hardness and ductility in the temperature range from around −140 to 130 °C, as well as increased resistance to chemicals, biological factors, and aging processes [10]. Because of its high reactivity and fast cure, pMDI has been extensively researched for its use as a UF and PF resin modifier, which resulted in the development of hybrid resins such as PF/pMDI and UF/pMDI, which were used in the studies on wood-based materials before [11,12]. The results of a few selected ones are summarized in Table 1.

As shown in Table 1, the introduction of pMDI to both UF and PF adhesive significantly improved the properties of commonly used wood-based materials such as particleboard, oriented strand board (OSB), and plywood. Interestingly, pMDI-modified adhesives were also found to be suitable for manufacturing composites from particles other than wood, in the case of which the stronger adhesion is needed to meet the standardized strength requirements. Studies have shown that hybrid adhesives worked well as binding agents for the production of boards made of, e.g., rape straw [21], sunflower husks [22], pine bark [23], waste corrugated paper [24], and kraft paper [25]. The outcomes have shown that, due to the application of PF/pMDI resin, produced composites were characterized by satisfactory mechanical and physical features, which allowed them to fulfill the requirements of proper standards.

An example of the potential use of PF/pMDI resin due to its high adhesion strength may be the production of particleboards with increased fire resistance. In general, the low fire safety of wood-based materials is usually considered a disadvantage, especially when used in interior or structural applications [26]. The presence of unprotected wooden elements may contribute to the spread of a fire and, therefore, as stated by Harada et al. [27], the following characteristics are expected from wood-based materials when used in constructions: they do not breakdown or deform in the presence of fire, the temperature of unexposed side does not exceed burning temperature of the entire material, and the structure of the material does not crack or become otherwise damaged because of the fire outside the building. To achieve these qualities, it is necessary to implement fire protection, for example, by treating the particles using fire retardants [28]. It protects the material throughout its entire cross-section, not only on the surface as in the case of fire-resistant coating application [29,30]. However, the impregnating solutions can not only influence the curing process of adhesives but also change the condition of the wood surface. According to Ayrilmis et al. [31], using impregnated wood can lead to the deterioration of resultant particleboard due to the change in the pH of wood, reduction in the number of hydroxyl groups available for bonding, and mechanical interference of salt. The outcomes of research regarding the production of boards from the particles impregnated with monoammonium phosphate, diammonium phosphate [32,33], Burnblock^®^ [34], borax, boric acid [35], and boric-acid–disodium-octaborate [36] confirm there negative influence. Therefore, it is necessary to develop a method of modifying resins to produce impregnated boards characterized by good strength properties.

Taking into account that PF/pMDI resin has not been used before for impregnated wood gluing, a project aimed at determining its suitability for the production of particleboard with increased fire resistance was started. This is the continuation of preliminary research that confirmed the effectiveness of fire protection of a mixture of potassium carbonate and urea and indicated favorable results in the case of modification of PF using 20% of pMDI [37]. However, considering the high cost of pMDI [38], the adverse impact of isocyanates on human health [39] and environmental aspects [40], there is a great need to optimize the loading of the modifier. Therefore, we decided to conduct research aimed at determining the effect of various amounts of pMDI on the properties of hybrid resins, which still has not been fully explained and described in the literature, especially in the case of small loadings, and to determine the optimal amount of pMDI that should be applied in the production of particleboards characterized by the increased resistance to fire.

## 2. Materials and Methods

### 2.1. Materials

Phenol-formaldehyde (PF) resin was provided by Silekol (Kędzierzyn-Koźle, Poland). Properties of applied unmodified phenolic resin can be found in Table 2. pMDI purchased from Bayer AG (Leverkusen, Germany) was characterized using the following characteristics: a solid content of 100%, chlorine hydrolytic of 96 mg/kg, NCO content of 32%, and a viscosity of 220 mPa·s. Reagents needed to prepare the impregnating solution such as potassium carbonate (99% pure, Altair Chimica, Saline, Italy) and urea (analytical purity, Chempur, Piekary Śląskie, Poland) were used as received without further purification. Pine (*Pinus sylvestris* L.) wood particles used in industrial conditions to produce the middle layer of three-layer particleboard were supplied by a local manufacturer of wood-based materials.

### 2.2. Determination of Properties of PF and PF/pMDI Adhesives

The adhesive formulations in this research included pure PF resin and PF resin with 5%, 10%, 15%, and 20% pMDI. After introducing the assumed modifier amount, the mixture was stirred manually until a proper homogenization was obtained.

The hybrid resins differing in the amount of pMDI we evaluated in terms of parameters commonly used to assess the quality of resins in the wood-based materials industry [41]. The solid content was determined according to EN 827 [42]. The viscosity of mixtures was determined using Brookfield DV-II+Pro viscometer (Middleboro, MA, USA). pH measurements were carried out using a Testo 206 pH-meter (Pruszków, Poland). Each test was repeated three times for every variant.

Thermogravimetric analysis (TGA) was used to study the thermal decomposition of the used formulations. The samples of 10 ± 0.2 mg were heated in the temperature range of 30–900 °C with a rate of 10 °C/min using a Netzsch TG209 F1 apparatus (Selb, Germany). The measurements were realized using Al_2_O_3_ crucibles in an inert atmosphere (nitrogen). The first mass derivative of the mass degradation (DTG) curves was calculated in reference to the obtained mass vs. temperature curves.

The thermal behavior during curing of the PF and PF/pMDI resins was analyzed using the differential scanning calorimetry (DSC) method. Samples of 20 ± 0.5 mg were placed in hermetic high-pressure crucibles at −10 °C to 280 °C with a rate of 10 °C/min. A Netzsch DSC 214 Nevio apparatus (Selb, Germany) and an inert nitrogen atmosphere were used.

### 2.3. Impregnation of Wood Particles and Particleboard Manufacturing

To determine the size distribution within the mixture of wood particles, the fractional composition was determined. The particles were passed three times through a set of flat sieves with the following square perforations: 0.315, 1.0, 1.5, 2.0, 2.5, 4.0, 5.0, and 6.3 mm. Based on the outcomes, it was found that the dimensions of the vast majority of particles were in the range between 1.0 and 2.5 mm.

The oven-dried particles characterized by a moisture content (MC) of 3 ± 2% were placed in 45 L HDPE containers filled with impregnating solution and soaked for 60 min at atmospheric pressure. A 30% aqueous solution of a mixture of potassium carbonate and urea in a weight ratio of 2:1 was applied for fire protection. Potassium carbonate was selected due to numerous favorable features such as excellent fire retarding efficiency, low iron corrosiveness, fungicidal properties, and no harm to human and animal health [43,44]. Moreover, urea included in the formulation of fire retardant has a synergistic effect on the kinetics of thermal decomposition and, thus, the effectiveness of fire protection [45]. After the assumed immersion time, particles were left to drain for 15 min and then dried in a laboratory oven to reach the MC of 4 ± 2%.

**Figure 1:**
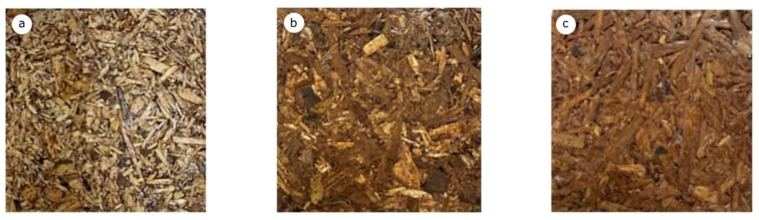
The appearance of particleboards containing various shares of impregnated particles: (**a**) 0% of impregnated particles; (**b**) 50% of impregnated particles; (**c**) 100% of impregnated particles.

Particleboard made of unimpregnated particles, bonded with pure PF adhesive was used as a reference variant for this research. Furthermore, the range of experimental variants assumed that four single-layer particleboards were produced for each adhesive formulation containing 0%, 5%, 10%, 15%, and 20% pMDI. Two of them were manufactured using 50% of impregnated particles and the remaining two were made entirely of impregnated particles, which allowed us to determine the optimal adhesive composition for two different shares of impregnated wood. The following parameters were used to produce the boards: assumed thickness of 10 mm, assumed density of 650 kg/m^3^, dimensions of 670 × 580 mm, gluing degree of 10%, pressing temperature of 180 °C, unit pressure of 2.5 N/mm^2^, and pressing time of 25 s/mm of the assumed board thickness. The appearance of the produced particleboards differing in the content of impregnated particles is shown in Figure 1.

### 2.4. Determination of Particleboards Properties

Produced particleboards were conditioned for seven days at relative humidity (RH) of 65 ± 5% and temperature of 21 ± 2 °C prior to testing. Determinations of the mechanical and physical properties were performed in accordance with relevant standards: density according to EN 323 [46], bending strength (MOR) and modulus of elasticity (MOE) according to EN 310 [47], internal bond (IB) according to EN 319 [48], internal bond after the boiling test (V100) according to EN 1087 [49], and thickness swelling (TS) after 24 h of soaking according to EN 317 [50]. The tests were carried out using 12 samples from each variant.

### 2.5. Statistical Analysis

Analysis of variance (ANOVA) was conducted to analyze the results of particleboard properties. Moreover, to distinguish homogeneous groups and assess the significance of observed changes, a HSD Tukey test at the significance level of α = 0.05 was performed using Statistica 13.3 software.

## 3. Results and Discussion

The results of the resin’s properties investigations are presented in Table 2. It was found that the modification of PF resin using pMDI led to the increase in solid content, viscosity, and pH of mixtures and the obtained values were higher by 12%, 78%, and 8% in the case of variants containing the highest loading of pMDI, respectively. The increase in viscosity of adhesives, which is a crucial parameter that significantly affects the mechanical performance of manufactured particleboard [51], was probably caused by the increase in solid content, reactions between pMDI and water, and the formation of urethane linkages between the PF resin and pMDI [19,52]. This phenomenon is consistent with the observations of Zheng et al. [53], who stated that, in the case of hybrid PF/pMDI resins, the viscosity increases with the increasing content of isocyanate up to 50%.

**Figure 2:**
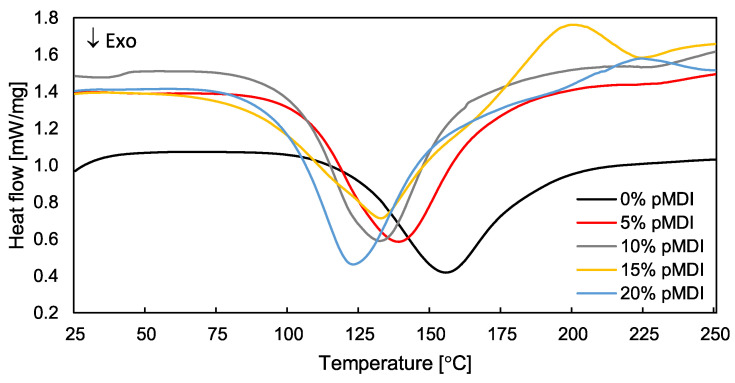
DSC curves of PF resin with the addition of different amount of pMDI.

The thermograms presented in Figure 2 illustrate the effect of the addition of various amounts of pMDI on the condensation process of PF resin, which is represented by an exothermic region with a single peak. Moreover, Table 3 summarizes the parameters characterizing the curing process of modified adhesives, i.e., onset temperature (T_onset_), peak temperature (T_p_), final temperature (T_endset_), and total heat (ΔH).

It was found that the condensation of hybrid PF/pMDI resin is a complex process, where the content of introduced pMDI plays a significant role. As the amount of pMDI increased, clear changes in the reaction kinetics were observed, confirming the modifier’s significant impact on the behavior of PF resin. The results have shown that, as the amount of pMDI increased, the exothermic peak temperature gradually decreased. In the case of maximum loading of isocyanate, the T_p_ was reduced by 32 °C compared to pure PF resin. Moreover, an increase in the values of total heat released during the process was also observed. In the case of pure PF resin, the ΔH value was 178.1 J/g and the addition of pMDI in the amount of up to 10% caused a gradual increase in the enthalpy value up to 278 J/g. However, a further increase in the loading of pMDI reduced the enthalpy to 190.2 J/g, which is still a higher value than pure PF resin. The decrease in T_p_ together with the increased heat release during the exothermic transition of the resin indicate the increased reactivity of the modified mixtures and the possibility of lowering the curing temperature of the experimental, hybrid adhesives. From a practical point of view, this modification may result in lower energy consumption during the technological process and, as a result, could contribute to reducing production costs and more efficient use of resources [54]. Moreover, considering that the reaction enthalpy is directly related to the conversion degree of the resin, it can be concluded that adding pMDI to PF resin allowed a higher conversion degree [55,56]. Observed improvement in the reactivity of PF resin, which is shown mainly by the reduction in T_p_, results from the fact that, in addition to the typical slow condensation reaction leading to the formation of methylene bonds (-CH_2_-), a second reaction takes place (almost simultaneously) as well. The reaction of the hydroxyl groups of PF resin with the isocyanate groups of pMDI leads to the formation of durable urethane linkages (-NH-CO-O-) [57,58,59,60]. The reaction of pMDI with the functional groups of PF resin is probably hindered by the considerable amount of water introduced with the PF resin and released during its condensation. As a result, it also disrupts the curing process. In turn, at the higher concentration of pMDI (20%), the formation of urethane linkages intensified which led to the formation of a larger number of urethane bonds and changed the reaction kinetics. It is also shown by the further decrease in T_p_ and increase in both T_onset_ and T_endset_, at which the exothermic transition begins and ends. Mixing pMDI with PF resin produced an oil–water emulsion, where pMDI created a dispersed phase of vitrified urea/urethane/biuret structures which accelerated the curing process of the PF/pMDI mixture [53,58].

**Figure 3:**
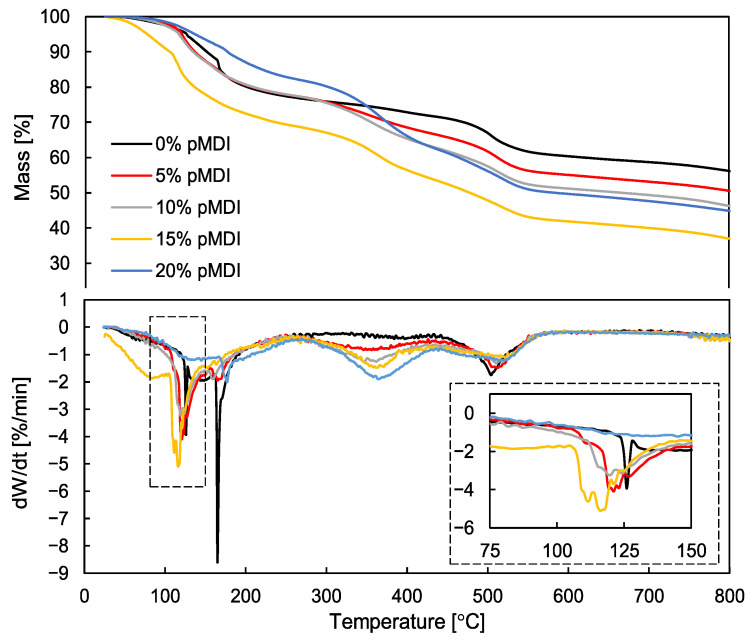
TG and DTG curves of PF/pMDI resins.

Despite the recorded improvement in the reactivity of PF/pMDI hybrid resin, we also noticed that the introduction of pMDI in the amount ranging from 5 to 15% resulted in a gradual decrease in resin’s thermal stability, especially at temperatures exceeding 300 °C (Figure 3, Table 4).

Overall, the thermal degradation process of PF resin includes three main degradation stages: post-curing, thermal reforming, and ring stripping [60,61]. Based on the analysis of TG/DTG curves, three mass-loss stages can be noted: a first in the temperature range up to 270 °C, a second between 270 and 450 °C, and a third between 450 and 550 °C. In the first stage of pyrolysis, usually occurring below 270 °C, a mass loss mainly related to the release of free formaldehyde, phenol, oligomer, and water introduced with PF resin and resulting from the progressing cross-linking reaction can be observed. In the second stage, recorded mass loss resulted from the decomposition of bridged methylene (thermal reforming). Furthermore, when the temperature exceeded 450 °C (third stage), the mass loss was caused by the degradation of methylene bonds, resulting in carbon monoxide and methane volatilization. Additionally, phenol was further degraded to the carbon structure at a temperature of about 500–550 °C [62,63,64], and the aromatic structure was degraded as well. Based on the analysis of TG/DTG curves and the data presented in Table 4, it can be observed that, in the first stage of pyrolysis, the addition of pMDI to PF resin in the amounts of 5 and 10% caused a slight shift of the maximum mass loss rate towards lower temperatures, while the residual mass (RM) values remained similar. The increase in the loading of pMDI to 15% resulted in a slight increase in mass loss by approx. 9% compared to the reference sample. More significant mass losses observed in the case of these variants were noted only in the next stage of degradation. It seemed that small content of pMDI (5–15%) caused the gradual loss of thermal stability of hybrid resin which has been shown by the decrease in the residual mass values. It should be also noted that, as the loading of pMDI increased, the maximum mass loss rate shifted towards higher temperatures. However, the thermal stability of the resin modified using 20% of pMDI seemed different. In this case, the addition of pMDI increased thermal stability of the resin in the temperature range up to approx. 350 °C, allowed us to obtain the residual mass at a level comparable to or slightly higher than noted for pure PF resin and to shift the maximum mass loss rate towards higher temperatures. In the next stages, the mixture containing 20% of pMDI also experienced a significant mass loss; however, the residual mass was slightly higher than observed for resins enhanced with smaller amounts of pMDI but still lower than in the case of pure PF resin. It is worth emphasizing that the results of TG/DTG are consistent with the outcomes obtained by Liu et al. [60] who showed that the mixture of PF resin with pMDI in a 1:1 weight ratio was characterized by better thermal properties than pure PF resin at temperatures below 500 °C. Reduced thermal stability of PF/pMDI resins containing small amounts of pMDI (5–15%) was probably related to the fact that, with such small loadings of isocyanate, the dominant reaction was slow condensation of PF resin [52]. The second reaction was between pMDI and hydroxyl of the methylol group (-CH_2_OH) of resol PF with the formation of a relatively small number of urethane linkages. As already mentioned, pMDI introduced into the system does not react with water to the same extent as with the hydroxyl groups of PF resin, which, in turn, may interfere with the formation of PF/pMDI structure and can lead to a reduction in thermal stability [52,58,65]. Furthermore, the addition of 20% pMDI to PF resin contributed to the formation of a larger number of more durable urethane bonds and, as a result, thermal stability at the temperature of up to 350 °C was improved. When the temperature rose, the thermal degradation process started for both PF resin and pMDI, which led to decreased thermal stability of PF/pMDI. According to Xu et al. [65], who obtained similar results in the case of MUF/pMDI resin, this effect can be attributed to the increased rigidity of the polymer molecules using aromatic pMDI with the bulky double phenyl ring.

**Figure 4:**
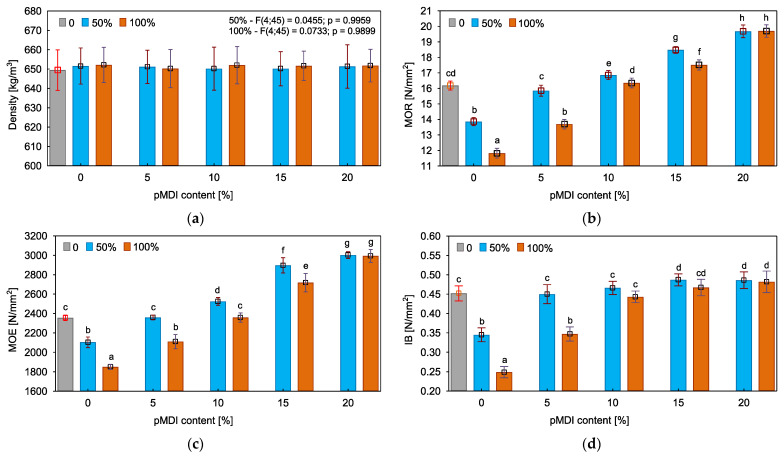
Properties of manufactured particleboard: (**a**) Density; (**b**) Bending strength (MOR); (**c**) Modulus of elasticity (MOE); (**d**) Internal bond (IB). (a–h letters indicate homogeneous groups).

Figure 4 presents the results of manufactured particleboards’ density and mechanical properties. The strength of produced materials is especially important because, even if the board achieves effective protection against fire, the deterioration caused by the treatment can still be a limiting factor in some applications, e.g., for structural purposes [66].

Based on the outcomes of the density measurements, it was found that both wood impregnation and the introduction of pMDI to the adhesive mixtures did not affect the obtained results. The average values ranged from 631.8 to 671.2 kg/m^3^, relatively close to the assumed one. Therefore, the effect observed by Du and Song [32], consisting of the increase in density of the board due to the use of fire retardant-treated particles, did not occur in this study. The comparison of variants bonded using unmodified PF resin showed that impregnation negatively impacted the strength of particleboards and, moreover, the greater the share of impregnated particles was, the more pronounced deterioration was. The results of bending strength, modulus of elasticity and internal bond were decreased by 18%, 11%, and 22% when the share of impregnated particles was 50% and by 27%, 21%, and 43% in the case of boards made entirely of impregnated wood. Studies have also shown that the introduction of pMDI to PF resin contributed to the improvement in the mechanical characteristics of particleboards. The improvement in adhesion strength probably resulted from the formation of crosslinking and/or linear structures (urea/biuret/dimmer/trimer), which was caused by the reactions between pMDI and water stored in the middle lamellae of wood cell walls [67]. Furthermore, pMDI can contribute to the improvement in adhesion due to the formation of polyurethane structures resulting from the reactions with hydroxyl groups of polysaccharides and phenolic groups of lignin contained in the wood tissue [17]. As stated by Zheng et al. [53], the introduction of isocyanate to PF resin could enhance the morphology of cured adhesive and, consequently, could toughen the resultant bond lines which may also have a favorable effect on the strength of the boards. The statistical analysis showed that, to achieve results as good as in the case of the reference variant, the amount of 5% and 10% should be applied for boards containing 50% and 100% of impregnated particles, respectively. Considering that in a previously conducted study only the addition of 20% was used [37], this research showed that reducing this loading by 75% or 50% is possible, depending on the share of treated particles.

Optimization of the amount of introduced pMDI is crucial due to the health risks, environmental aspects, and high price [10]. Many diisocyanates such as, for example, methylene diphenyl diisocyanate (MDI) and toluene diisocyanate (TDI) are classified as substances suspected of causing cancer (H351). Moreover, Husskonen et al. [68] stated that diisocyanates generally act as eye, respiratory, and skin irritants. The estimated number of incidents of occupational asthma related to their use in the European Union ranges from 2350 to 10,150 cases each year and the production of adhesives is identified as a particularly exposed branch of industry [69]. On the industrial scale, diisocyanates are usually produced from petroleum resources and, in most cases, they are obtained by reacting a primary amine with highly toxic phosgene [70,71]. The exposure of the natural environment to isocyanates can lead to the pollution of water [72], soil [73], and air [74]. Therefore, the content of pMDI in hybrid resins should be adjusted to be as low as possible to decrease the negative impact and reduce production costs.

**Figure 5:**
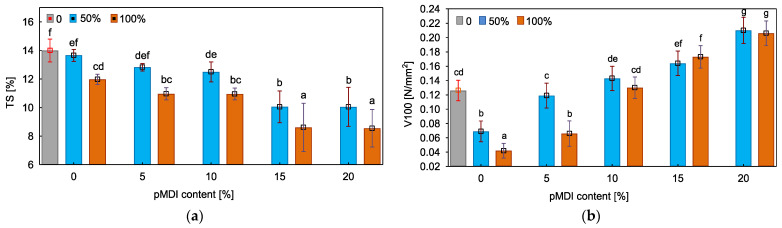
Water resistance of particleboards: (**a**) Thickness swelling (TS); (**b**) Internal bond after boiling (V100). (a–g, letters indicate homogeneous groups).

Figure 5 presents the results of thickness swelling and internal bond after boiling, indicating the water resistance of manufactured particleboards. Taking into account the potential structural application of produced materials, it is essential to investigate their behavior under the conditions of constant or periodic exposure to water usually resulting in the creation of internal stresses and deterioration in the strength of adhesive bonds [75,76].

In the case of boards bonded using pure PF resin, the results have shown that adding fire-retardant-treated particles in the amount of 50% did not affect the results of thickness swelling. However, when the share of impregnated particles increased to 100%, manufactured particleboards demonstrated lower swelling values. According to Jayamani et al. [77], impregnation of wood with potassium carbonate reduces its hydrophilicity due to ongoing reactions between fire retardant and hydroxyl groups of wood and changes in the orientation polarization [78]. Furthermore, the reason for such an effect could be a partial degradation of hemicelluloses which was observed before for wood treated using potassium carbonate as well [77]. On the other hand, in the case of boards bonded using unmodified PF resin, the results of the internal bond after boiling have shown a deterioration of 45% and 67% due to the use of impregnated particles in the amount of 50% and 100%, respectively. Based on the statistical analysis, it was found that, to produce boards with the same properties as the reference variant, the PF resin should be modified using 5% pMDI in the case of 50% impregnated particles and 10% pMDI in the case of 100% impregnated particles. Overall, the introduction of pMDI to the PF resin led to an improvement in the particleboard’s resistance to water and as the content of isocyanate increased, the observed improvement was more noticeable. According to Iswanto et al. [79], the favorable effect of pMDI on TS and V100 could result from its reaction with hydroxyl groups of wood constituents which reduces the accessibility of water. Moreover, the access of water could also be decreased by the improvement in morphology of bond lines which prevents water penetration into the joint [53,80,81].

Based on the results of all properties, it was found that, to achieve properties as good as the reference variant, the optimal content of pMDI for 50% of impregnated particles is 5%, and for 100% of impregnated particles it is 10%. The boards produced this way were classified as P4 boards (load-bearing boards for use in dry conditions) according to EN 312 [82]. The outcomes have also shown that to achieve the properties of P5 boards (load-bearing boards for use in humid conditions), the loading of pMDI should be increased to 15% for both shares of impregnated particles.

The results of ANOVA are presented in Table 5. Based on statistical parameters, a significant influence of both the share of impregnated particles (factor A) and the amount of pMDI introduced to PF resin (factor B) on the values of all parameters investigated was confirmed. This is evidenced by the high values of the sum of squares (SS), mean square (MS) and Fisher statistic (F), and low values of *p*-value (*p* < 0.05). Moreover, there is a close interaction between factors A and B which means that the effect of one factor depends on the level of the other factor; the SS, MS, and F values obtained for this interaction are also statistically significant.

## 4. Conclusions

The obtained results indicate that hybrid PF/pMDI resin is suitable for gluing wood particles impregnated with a mixture of potassium carbonate and urea in particleboard production.

During the course of the research, the follow has been demonstrated:

- As the amount of pMDI increases, an increase in viscosity, solid content, and pH of the adhesive mixtures can be observed.
- The modification of PF resin using pMDI positively affects the condensation kinetics and reactivity of adhesive mixtures, as evidenced by a decrease in peak temperature and an increase in total heat release.
- The addition of pMDI in the range of 5% to 15% leads to a gradual decrease in the thermal stability of hybrid resins at temperatures above 300 °C. Further increasing the pMDI content to 20% contributes to an enhancement in resin thermostability in the temperature range up to 350 °C.
- Impregnation of wood particles does not affect the density of resultant particleboards, regardless of the share of fire retardant-treated wood. However, their mechanical properties, such as bending strength, modulus of elasticity and internal bond, deteriorate in the case of boards bonded using neat PF resin.
- The enhancement of PF resin using pMDI results in improved mechanical properties and water resistance of these boards and allows the production of materials with properties as good as the untreated board.
- The loading of pMDI was optimized to be 5% in the case of particleboards containing 50% impregnated particles and 10% for boards produced using 100% impregnated wood. These materials can be classified as P4 particleboards; to upgrade the class to P5, the loading of pMDI should be increased to 15%.

## Figures and Tables

**Table 1:** Examples of studies on using pMDI-modified hybrid resins for the production of wood-based materials.

Type of Hybrid Resin	Type of Wood-Based Material	Effect of pMDI Addition	References
UF/pMDI	Particleboard	Improved mechanical properties; Improved water resistance; Reduced pressing time; Reduced formaldehyde emissions.	[13]
	Plywood	Improved bonding quality; Improved bending strength and modulus of elasticity; Reduced pressing time; Reduced formaldehyde emissions.	[14,15,16]
PF/pMDI	Particleboard	Improved mechanical properties; Improved water resistance; Reduced formaldehyde emissions; Improved screw holding strength.	[17]
	Oriented strand board	Improved mechanical properties; Improved water resistance.	[18]
	Plywood	Improved bonding quality; Improved bending strength and modulus of elasticity; Reduced pressing time.	[19,20]

**Table 2:** Properties of PF/pMDI resins. (a–e letters indicate homogeneous groups).

pMDI Content (%)	Solid Content (%)	Viscosity (mPa·s)	pH
0	55.41 ^a^ ± 0.13	643 ^a^ ± 21.2	9.13 ^a^ ± 0.08
5	57.24 ^b^ ± 0.19	751 ^b^ ± 21.4	9.33 ^b^ ± 0.05
10	58.97 ^c^ ± 0.08	887 ^c^ ± 16.4	9.51 ^c^ ± 0.02
15	60.03 ^d^ ± 0.07	1045 ^d^ ± 26.7	9.70 ^d^ ± 0.04
20	62.09 ^e^ ± 0.22	1146 ^e^ ± 15.3	9.81 ^e^ ± 0.02

**Table 3:** Results of DSC analysis of PF resin with the addition of various amounts of pMDI.

pMDI Content (%)	T_onset_ (°C)	T_p_ (°C)	T_endset_ (°C)	ΔH (J/g)
0	118.4	155.4	186.4	178.1
5	104.1	138.5	169.2	249.2
10	101.4	132.2	163.0	278.8
15	88.0	132.4	133.7	227.3
20	96.4	122.8	149.4	190.2

**Table 4:** Parameters characterizing thermal degradation of PF/pMDI resins.

pMDI Content (%)	1st Stage				2nd Stage		3rd Stage		RM (%)
	<270 °C				270–450 °C		450–550 °C		
	T_p1_ (°C)	Mass _Tp1_ (%)	T_p2_ (°C)	Mass _Tp2_ (%)	T_p3_ (°C)	Mass _Tp3_ (%)	T_p4_ (°C)	Mass _Tp4_ (%)	
0	126.1	94.9	165.1	86.9	346.9	74.7	504.3	66.2	53.1
5	121.1	94.4	164.9	84.8	346.4	72.5	512.7	60.0	47.8
10	119.6	94.0	159.1	85.5	347.5	71.0	510.6	56.3	41.5
15	115.8	86.0	154.0	77.2	361.7	60.9	521.8	45.5	32.9
20	145.0	93.9	176.7	89.9	364.4	72.0	515.1	54.4	41.9

**Table 5:** The results of the ANOVA.

Main Factor	Statistical Parameters				
	*SS*	*Df*	*MS*	*F*	*p*
MOR					
A	26,865.64	2	13,432.82	131,006.7	0.00
B	5840.87	1	5840.87	56,964.4	0.00
A × B	15,320.59	5	3064.12	29,883.5	0.00
MOE					
A	621,561,459	2	310,780,730	97,600.05	0.00
B	132,690,888	1	132,690,888	41,671.30	0.00
A × B	342,785,889	5	68,557,178	21,530.24	0.00
IB					
A	17.89	2	8.94	22,948.34	0.00
B	3.65	1	3.65	9358.69	0.00
A × B	11.27	5	2.25	5784.40	0.00
TS					
A	12,194.12	2	6097.06	7048.98	0.00
B	5233.27	1	5233.27	6060.33	0.00
A × B	6505.02	5	1301.00	1504.13	0.00
V100					
A	1.76	2	0.88	3431.82	0.00
B	0.19	1	0.19	732.06	0.00
A × B	1.23	5	0.24	956.69	0.00

## Data Availability

The data presented in this study are available on request from the corresponding author.
